# Therapeutic, Prophylactic, and Functional Use of Probiotics: A Current Perspective

**DOI:** 10.3389/fmicb.2020.562048

**Published:** 2020-09-11

**Authors:** Maria Aponte, Nicoletta Murru, Mahtab Shoukat

**Affiliations:** ^1^Department of Agricultural Sciences, University of Naples Federico II, Portici, Italy; ^2^Department of Veterinary Medicine and Animal Production, University of Naples Federico II, Naples, Italy

**Keywords:** probiotics, health beneficial effects, host–microbe interactions, immunomodulation, gut microbiota, dysbiosis

## Abstract

Probiotics are considered as the twenty-first century panpharmacon due to their competent remedial power to cure from gastrointestinal dysbiosis, systematic metabolic diseases, and genetic impairments up to complicated neurodegenerative disorders. They paved the way for an innovative managing of various severe diseases through palatable food products. The probiotics’ role as a “bio-therapy” increased their significance in food and medicine due to many competitive advantages over traditional treatment therapies. Their prophylactic and therapeutic potential has been assessed through hundreds of preclinical and clinical studies. In addition, the food industry employs probiotics as functional and nutraceutical ingredients to enhance the added value of food product in terms of increased health benefits. However, regardless of promising health-boosting effects, the probiotics’ efficacy still needs an in-depth understanding of systematic mechanisms and factors supporting the healthy actions.

## Introduction

The probiotic concept was first introduced by the Nobel Prize, Ilya Ilyich Mechnikov in 1908 during his study about yogurt-derived health-boosting effects on Bulgarian peasants (Kareb and Aïder, 2019). The word probiotic originates from the Greek word “pro-bios,” which means “for life.” In 2001, the Food and Agriculture Organization of the United Nations (FAO) and the Health Organization (WHO) provided a collaborative and comprehensive probiotics’ definition as “*Live microorganisms, which when administered in adequate amounts; exert a beneficial effect on the host’s health.”* However, a slight modification in probiotics definition is accomplished by the International Scientific Association for Probiotics and Prebiotics (ISAPP) as *“Live microorganisms that, when administered in adequate amounts, confer a health benefit on the host”* (Hill et al., 2014). Probiotics mostly belong to Gram-positive bacteria, having natural habitat in the human’s gut. Probiotics may alleviate gastrointestinal dysbiosis, lower serum cholesterol, ameliorate cancer, and prevent allergic and autoimmune disorders (Hajavi et al., 2019).

*Lactobacillus*, *Bifidobacterium*, *Streptococcus*, *Saccharomyces*, *Bacillus*, and *Enterococcus* are the most popular probiotics genus sources, and their application as probiotics are extensive in fermented food products, non-fermented food items, as well as functional and nutraceutical dietary supplements (Di Cerbo et al., 2016). Because of the various health claims, probiotics-based food currently occupies a considerable number of market shelves. As a matter of fact, nearly 2,000 clinical research studies regarding various prophylactic and therapeutic health benefits related to probiotics have been currently published. In addition, outstanding pace was observed toward exploring various molecular mechanisms by which probiotics interact with host and exert their impact on the target cells. However, there is still a gap when translating the several recognized probiotics’ health benefits mechanisms into target therapies with specific probiotics (Kleerebezem et al., 2019).

The probiotics’ efficiency may be compromised by several factors: an adequate delivery system is one of them. Food processing and storage conditions (water activity, temperature, pH, oxidation, osmotic pressure, etc.), coupled with the survival through the hostile gastrointestinal tract (GIT) environment (acidic pH, bile salts, digestive enzymes, etc.) and the ability to colonize the gut, are the major challenges (Li et al., 2019a). However, microencapsulation seems to be a highly productive and holistic approach to overcome this problem (Feng et al., 2020).

Probiotics functionality is mainly based on bacterial interaction with host gut microbiota through a number of actions including (Sharma, 2019):

• enhancing the intestinal barrier function by regulating proinflammatory cytokines and chemokines’ profile;
• improving the intestinal barrier selectivity through higher mucin, immunoglobulin A (IgA), and defensins production;
• increasing the production of vitamins, minerals, short-chain fatty acids (SCFAs), and growth regulators to strengthen the intestinal epithelium layer;
• promoting antiangiogenic factors, cytokines (IL-2 and IL-12), and antioxidants;
• reducing the intestinal pH;
• increasing anti-inflammatory molecules formation to enhance the immune system;
• modifying the intestinal microbiota;
• regulating mechanisms of apoptosis and cell differentiation;
• retarding tyrosine kinase and other deleterious pathways.

Recent preclinical and clinical trials highlighted the significance of probiotics’ intake in the prevention and treatment of gastrointestinal diseases [salmonellosis, *traveler*’s diarrhea, antibiotic-associated diarrhea (AAD), acute diarrhea, *Helicobacter pylori* gastroenteritis, irritable bowel syndrome (IBS), inflammatory bowel diseases (IBDs), hyperlipidemia and hypercholesterolemia (Kim et al., 2017), cancer (Vivarelli et al., 2019), lactose intolerance (LI) (Vitellio et al., 2019), autoimmune diseases (Dwivedi et al., 2016), as well as bone (Nath et al., 2018), neurodegenerative (Li W. et al., 2020), and metabolic disorders (León et al., 2019)].

## Health-Boosting Spectrum of Probiotics

### Lactose Intolerance

Lactose intolerance is a genetically determined β-galactosidase deficiency resulting in the inability to hydrolyze lactose into the monosaccharides glucose and galactose. Unabsorbed lactose is then metabolized by colonic bacteria (Oak and Jha, 2019). Lactase activity is age dependent, as it declines with age. Symptoms of LI include diarrhea, abdominal discomfort, and flatulence after consumption of milk and dairy products (Syngai et al., 2016). The consumption of *Streptococcus thermophilus* and *Lactobacillus delbrueckii* subsp. *bulgaricus* in yogurt proved to be helpful in alleviating LI, partly because of the higher beta-galactosidase activity (Kechagia et al., 2013). However, a number of studies demonstrated that milk containing *Bifidobacterium longum* and *Lactobacillus acidophilus* can be easily tolerated by LI individuals (Vonk et al., 2012). Gingold-Belfer et al. (2020) carried out a pilot study to assess the efficacy of probiotics with β-galactosidase activity on symptoms of lactose malabsorption by means of hydrogen breadth test (LHBT). Eight female patients with positive LHBT were included in the study and treated for 6 weeks with probiotics. According to outcomes, in more than 50% of cases, symptoms’ severity or frequency decreased, as even revealed by the LBHT normalization. Furthermore, the use of a novel formulation, containing *B. longum* BB536 and *Lactobacillus rhamnosus* HN001 plus vitamin B6, in 23 LI subjects with persistent symptoms proved to be useful in alleviating the condition through a positive modulation of the gut microbial composition (Vitellio et al., 2019). Similarly, the administration of two new probiotic strains, i.e., *Bifidobacterium animalis* subsp. *animalis* IM386 and *Lactobacillus plantarum* MP2026 to 44 LI subjects for 6 weeks, significantly lowered the diarrhea and flatulence symptoms (Roškar et al., 2017) (Table 1).

**TABLE 1:** Probiotics against LI disorder: a list of the main available *in vivo* trials.

**No of participants**	**Probiotic strain**	**Therapeutic effect**	**References**
44	*B. animalis* subsp. *animalis* IM386 and *L*. *plantarum* MP2026	Significant lowering effect on diarrhea	Roškar et al. (2017)
23	*B*. *longum* and *L*. *rhamnosus* with vitamin B6	Alleviation in LI symptoms	Vitellio et al. (2019)
60	*L. reuteri* and tilactase	Improved lactose digestion	Ojetti et al. (2010)
27	*L. casei* and *B. breve*	Decreased H_2_ production intake in lactose-intolerant patients	Almeida et al. (2012)
30	*L. acidophilus* DDS-1	Improved abdominal symptoms	Pakdaman et al. (2016)

### Gastrointestinal Tract Disorders

Probiotics have been shown to promote health benefits in many diseases related to an unbalanced GIT microbiota. Scientific evidences about probiotics’ health benefits in alleviating the symptoms of several GIT diseases are well documented (Ritchie and Romanuk, 2012). This section summarizes the main GIT diseases for which probiotics’ restoration of the microbial balance proved to be useful (Table 2).

**TABLE 2:** *In vitro* and *in vivo* effect of probiotics against gastrointestinal tract (GIT) diseases.

**Disease**	**Study model**	**Probiotic strain**	**Therapeutic effect**	**References**
Antibiotic-associated diarrhea	*In vivo*	*L. plantarum* DSM 9843 (LP299V)	No significant effect on the abdominal symptoms (pain, vomiting, flatulence, and distension)	Olek et al. (2017)
	*In vivo*	Yogurt combination of LGG, La-5 and Bb-12	Mild symptoms in the probiotic group	Fox et al. (2015)
	*In vivo*	*L. acidophilus* CL1285 and *L. casei* LBC80R	Decreased incidence of AAD	Gao et al. (2010)
Inflammatory bowel disease	*In vivo*	*L. salivarius*, *L. acidophilus*, and *B. bifidum* strain BGN4	Long time period intervention is more effective to alleviate IBD	Palumbo et al. (2016)
	*In vivo*	VSL#3	About 60% (61.8) of patients assumed all the prescribed probiotic therapy in a real-life setting, despite a good safety profile	Sivamaruthi (2018)
	*In vivo*	Lactobacilli, Bifidobacteria, Enterococci, Streptococci, and Bacilli	Majority of trials reported preventive effects	Clarke et al. (2012)
*H. pylori* infection	*In vitro*	*L. rhamnosus* and *L. acidophilus*	Significant decrease in infection	Chen et al. (2019)
	*In vivo*	*L. rhamnosus* and *L. acidophilus*	Anti-inflammatory activity	Chen et al. (2019)
	*In vivo*	*S. boulardii*	Improved calories-tolerance and reduced diarrhea	Czerucka et al. (2007)
	*In vivo*	*S. boulardii*	Decreased severity and duration of infectious diarrhea in children	Nami et al. (2015)
	*In vitro*	*S. cerevisiae* IFST062013 isolated from fruit juice	Significant antibacterial effects against Gram-negative bacteria and improved lymphocyte proliferation and cytokine production in treated mice	Fakruddin et al. (2017)

#### Antibiotic-Associated Diarrhea

Antibiotic-associated diarrhea is a condition characterized by disturbance in endogenous GIT microbiota following antimicrobial therapies. Heavy antibiotic treatments may cause mild or severe episodes of diarrhea by suppressing the normal microflora and by stimulating the overgrowth of opportunistic or pathogenic strains. AAD related to the overgrowth of *Clostridium difficile* is also termed as *C. difficile*-associated diarrhea (Sanchez et al., 2017). However, the use of *Leuconostoc cremoris* and of *Bacillus*, *Bifidobacterium*, *Lactobacillus*, *Lactococcus*, *Saccharomyces*, or *Streptococcus* spp.—individually or in combination—has been proved to have a protective effect in the treatment of AAD (Syngai et al., 2016). Alberda et al. (2018) demonstrated that the administration of *Lactobacillus casei* drink in an intensive care unit provided a noticeable preventative effect for AAD as well as *C. difficile*-associated diarrhea. According to a recent meta-analysis, in people treated with probiotics, AAD appeared in 8.0% of the cases, against the 17.7% of the control group. Data from 17 studies, for a total of 3,631 patients, demonstrated that use of probiotics can reduce the risk of AAD by 51% (Blaabjerg et al., 2017). More recently, Esposito et al. (2018) evaluated the efficacy of *Lactobacillus rhamnosus* (LGG), AAD in children with hypospadias repair. In the randomized controlled trial (RCT), *L. rhamnosus* GG associated with antibiotics significantly reduced the incidence and the duration of postoperative AAD and the incidence of postoperative complications, such as urethral fistula and foreskin dehiscence.

#### Traveler’s Diarrhea

According to epidemiological data, 20–60% of people suffers from traveler’s diarrhea around the world, usually following transferring from industrialized to developing countries. This condition is associated with the passage of three or more unformed stools per day coupled with symptoms such as abdominal pain or cramps. Bacteria are the most common cause in 60–85% of cases, and the usually pointed out pathogen is *Escherichia coli* followed by *Campylobacter jejuni*, *Shigella*, and *Salmonella* spp. (Giddings et al., 2016). According to an adaptive meta-analysis based on double-blind RCTs, probiotics exhibited a statistically significant efficacy in the prevention of traveler’s diarrhea (Bae, 2018). Sniffen et al. (2018) noted that it is important to properly select the probiotic strain(s), matching with the targeted disease and condition. In detail, *Saccharomyces boulardii* CNCM I-745 was found to be more beneficial on bacterial diarrhea, while *L. rhamnosus* GG (LGG) showed beneficial effects against viral and idiopathic diarrhea (Sniffen et al., 2018).

#### Inflammatory Bowel Diseases

Inflammatory bowel disease is a chronic, multifactorial disorder caused by inflammation of the GIT that may induce severe watery and bloody diarrhea accompanied by abdominal pain. IBD is a broader term and includes ulcerative colitis (UC), Crohn’s disease (CD), and pouchitis affecting both colon and small intestine (Derwa et al., 2017). Genetic and environmental factors, dysregulation of immune system, intestinal dysbiosis, and oxidative stresses are reported as key factors for the IBD onset (Moeinian et al., 2013). Probiotics have been proven to exert an actual influence toward both the GIT microbiota and the immune system (Di Cerbo et al., 2016). The multiple strains formulation VSL#3—eight probiotic strains plus *E. coli* Nissle 1917—proved to be effective in the prevention of IBD (Jia K. et al., 2018). In a double−blind, placebo−controlled study with 107 patients, the BIO−25 capsules (a combination of 11 probiotic strains belonging to *Lactobacillus*, *Bifidobacterium*, *Streptococcus*, and *Lactococcus* spp. genera) administration twice daily for 8 weeks did not result in significant fecal microbial changes. Response to therapy was associated with a reduction in *Bilophila* and increased baseline proportions of bacterial genera such as *Faecalibacterium* and *Ruminococcus* spp. (Hod et al., 2018). Evidence collected with reference to CD do not recommend probiotics; indeed, systematic surveys are scarce and mostly refer to the pediatric age. On the other hand, for UC treatment, some clinical trials support efficacy, especially when multistrain probiotics are used (Guandalini and Sansotta, 2019). Probiotics may exert therapeutic effects toward IBD condition by affecting the composition of the microbial ecosystem, by competing for nutrients and adhesion sites, via cell–cell communication, and by producing antimicrobial substances. In addition, probiotics interact with the host immune system through bacterial products, cell wall components, or DNA (Jonkers et al., 2012). Until now, no considerable adverse effect of the probiotic intervention on IBD patients has been reported except one UC patient who suffered from mild dry cough in the case of *B. longum* 536 supplementation (Sivamaruthi, 2018).

#### Irritable Bowel Syndrome

Irritable bowel syndrome is a chronic, heterogeneous condition often characterized by abdominal pain, bloating, and altered bowel habits (constipation, diarrhea, or alternation of both) (Barbara et al., 2016). The main pathophysiological factors leading to IBS are psychological (stress and emotional status), social (upbringing and support system), and biological (gut motility and visceral sensitivity), which interact in a complex way to exacerbate the symptoms (Lacy et al., 2016). Breast milk derived-*Lactobacillus gasseri* BNR17 mitigated diarrhea and other IBS symptoms in two independent RCTs (Kim et al., 2018; Shin et al., 2018). Dale et al. (2019), in a recent meta-analysis, reported that multistrain probiotic supplementation is more effective as compared to single strain for IBS symptoms alleviation. Similar findings were also mentioned in a further recent systematic meta-analysis, even if, according to the authors, no definitive conclusion can be derived (Ford et al., 2018).

#### *Helicobacter pylori* Infection

*Helicobacter pylori* is a widespread Gram-negative and spiral-shaped pathogen. It is the major causative agent of chronic gastritis and peptic ulcer, and it is a risk factor for gastric malignancies (Patel et al., 2014). Probiotic strains may reduce the rate of *H. pylori* by 5–10%, and it was found successful in the remission of the associated side effects (Alvi et al., 2016). The results of a double-blind placebo-controlled study suggested that *S. boulardii* and *Lactobacillus johnsonii* La1 can decrease *H. pylori* loads but are not able to induce a complete eradication of the pathogen (Homan and Orel, 2015). Furthermore, *S. boulardii* has been proven to improve the eradication rate if coupled to the standard *H. pylori* eradication therapeutic protocol. Further studies are needed in the case of *Lactobacillus reuteri*, as good results might be achieved (Homan and Orel, 2015). Different studies suggested that *H. pylori* eradication rate may be improved by a pretreatment with bifidobacterial-containing yogurt for 4 weeks (Sheu et al., 2006). Additionally, a network meta-analysis from 140 studies concluded that the eradication rate in the control (standard therapy alone) and in the probiotics-supplemented group was 70.5 and 84.1%, respectively. By contrast, adverse events rates were 30.1 and 14.4%, respectively (Wang et al., 2017). There is a robust evidence on supporting the use of probiotics in conjunction with antibiotics for the treatment of *H. pylori* infection. Nevertheless, optimal probiotic strain, dose, and treatment length are yet to be determined (Boltin, 2016).

### Disorders of Body Districts Other Than GIT

#### Respiratory Tract Infections

Respiratory tract infection (RTI) is a frequently occurring disease in humans, and a large range of etiological agents challenge the development of efficient therapies. Recent researches suggest that probiotics may decrease the risk or the duration of RTI symptoms. However, the antiviral mechanisms of probiotics are not clear yet (Lehtoranta et al., 2014).

Strain-specific effects of probiotics against RTI still have inadequate supporting data. This is evident by Laursen and Hojsak (2018) meta-analysis on 15 RCTs in which LGG and *B. animalis* subsp. *lactis* BB-12 were orally administered to daycare children. LGG significantly reduced RTI, while BB-12 did not show any noticeable results. Further findings about BB-12 probiotic strain efficacy for RTI treatments in children reported no considerable preventive effect (Hojsak et al., 2016). However, Rautava et al. (2008) reported that BB-12 combined with LGG substantially reduced the RTI recurrences in children. In another RCT open-label clinical study, yogurt added with probiotic *Lactobacillus paracasei* N1115, orally administered for 12 weeks to middle- and older-aged people, proved to be effective to mitigate RTI through increasing T cells (CD3+), which modulate the native immune system (Pu et al., 2017). By contrast, a meta-analysis of 21 RCTs reported insufficient evidence to support the therapeutic effect of probiotics against RTI, except for probiotic *L. casei* LCA (Amaral et al., 2017).

#### Urinary Tract Infections

Urinary tract infection (UTI) is more common among women compared to men and frequently tend to recur. *E. coli*, *Proteus*, *Klebsiella*, and *Staphylococcus* spp. are intestinal uropathogens generally considered as causative agents. The repletion of vaginal lactobacilli might be beneficial, as depletion can lead toward higher UTI risk (Fontana et al., 2013). It has been observed that high levels of vaginal colonization with probiotic *Lactobacillus crispatus* are associated with a significant reduction in recurrent UTI (Stapleton et al., 2011). A meta-analysis comparing the occurrence of recurrent UTI in 294 adult female patients across five studies showed no statistically significant difference in the risk for recurrent UTI in patients receiving probiotic lactobacilli versus controls. However, upon ineffective strains removal, a statistically significant decrease was found in recurrent UTI patients treated with probiotic lactobacilli (Grin et al., 2013). Wolff et al. (2019) conducted a double-blinded RCT to evaluate if oral probiotics could induce changes in the uropathogens/*Lactobacillus* (U/L) ratio among healthy premenopausal young women. Oral administration of *L. rhamnosus* GR-1 and *L. reuteri* RC-14 induced no considerable changes in U/L ratio in healthy female individuals. Sadeghi-Bojd et al. (2020) conducted RCTs among healthy children who recovered from first febrile UTI. Oral intervention with a probiotic mixture of *L. acidophilus*, *L. rhamnosus*, *Bifidobacterium bifidum*, and *Bifidobacterium lactis* was given for 18 months. Results indicated that *E. coli* and *Klebsiella pneumoniae* were the two core agents responsible for UTI recurrence in children, while probiotics reduced the UTI recurrence risk. Similar findings were also reported by Koradia et al. (2019) in a double-blind, placebo-controlled pilot efficacy trial based on Bio-Kult Pro-Cyan (a combination of cranberry extract and two lactobacilli strains) administration against recurrence of UTI in premenopausal mature women. A 26-week intervention illustrated a significant preventive action of Bio-Kult Pro-Cyan in UTI recurrence risk.

#### Chronic Kidney Disease

Chronic kidney disease (CKD) is a lethal global syndrome, also called “silent disease,” characterized by progressive reduction in renal functions, kidney infection commencement, and premature death. Incidence of diabetes mellitus, obesity, and hypertension largely contributed to the prevalence of CKD (Fagundes et al., 2018). Wang et al. (2015) reported prophylactic and therapeutic effects of *Lactobacillus* and *Bifidobacterium* spp. in peritoneal dialysis patients. Probiotics oral administration for 6 months considerably reduced the serum endotoxins and proinflammatory cytokines tumor necrosis factor alpha (TNF-α), interleukin (IL)-6, and IL-5, while it improved anti-inflammatory cytokine IL-10 activity. Considerable variations in the structure and functionality of gut microbiota between humans and animals with severe CKD were reported. The most evident effect is the abatement in the *Bifidobacterium*/*Enterobacteriaceae* ratio. In addition, many investigations asserted that CKD is associated with lower counts of *Bifidobacterium* and *Lactobacillus* spp., coupled with *Enterobacteriaceae* prevalence in the intestine (Sircana et al., 2019).

Guida et al. (2017) investigated the symbiotic effect of probiotics on plasma p-cresol concentration (uremia toxin) in kidney transplant recipients through an *in vivo* randomized trial. A 1 month trial demonstrated that probiotics could reduce the uremia toxin concentrations. Jia L. et al. (2018) carried out a meta-analysis of randomized controlled interventions in order to evaluate the efficacy of probiotic supplementations in adult CKD patients. Probiotics therapy could decrease p-cresyl sulfate, increase IL-6 levels, and improve the gut barrier function. However, blood urea nitrogen, hemoglobin, and serum creatinine concentrations remained constant, independently by the sample size and the poor number of studies. Similarly, another RCT-based meta-analysis of probiotics in CKD control revealed more promising results to manage urea levels in non-dialysis CKD patients. On the other hand, no significant change in glomerular filtration rate, uric acid, serum creatinine, and C-reactive protein concentrations were found (Tao et al., 2019).

#### Bacterial Vaginosis

Bacterial vaginosis (BV) is the predominant vaginal infection mainly occurring in early age female individuals, in which continuous off-white irregular vaginal discharge is the main symptom. Downfall growth of *Lactobacillus* species and surging overgrowth of vaginal anaerobic Gram-negative bacteria, i.e., *Gardnerella vaginalis*, lead to imbalance in the vaginal microecology (Masoudi et al., 2016). The pathological severity of BV is associated with elevated risks of preterm delivery, abrupt abortion, pelvic inflammation, and endometritis. *G. vaginalis* is the main pathogen producing polymicrobial biofilm, which acts as a binding agent against vaginal epithelial cells (Sabbatini et al., 2018).

*Lactobacillus*, *Bifidobacterium*, and *Saccharomyces* spp. have the potential to interfere with the pathological pattern of BV. Sabbatini et al. (2018) demonstrated the inhibitory response of probiotic *S. cerevisiae* against *G. vaginalis* infection in *in vivo* trials. Such preventive effects were based on different actions, such as amelioration of inflammatory cytokines, retard sialidase enzyme activity, and reduced *G. vaginalis* binding capacity to vaginal epithelial cells. In addition, in a recent test on mice, probiotic *Clostridium butyricum* WZ001 strain not only suppressed the growth of *G. vaginalis* and decreased inflammatory cytokines but also improved the lactobacilli growth to normalize the vaginal microecological balance (Zhou et al., 2019). A meta-analysis of probiotics-based RCTs for VB treatment reported that currently combo treatments strategies, i.e., probiotics plus antibiotics, are more effective than single applications of both. Moreover, probiotics are useful and effective against BV infection, but efficacy is not clear due to quality and heterogeneity of clinical trials (Li et al., 2019b).

#### Androgenetic Alopecia

Under normal conditions, 50–100 hairs fall from humans daily. However, if hair fall is >100 hairs/day, then it is a symptom of alopecia disorders. Hair loss is a multifactorial disease induced by hormonal disturbance, psychological instability (dementia, depression, and stress), poor nutrition, dandruff issue, and chemotherapy (Choi et al., 2015). 5-α reductase inhibitors are commonly used to cope with this disorder, particularly in men. However, various preclinical and clinical interventions model studies pointed out a potential therapeutic role of probiotic supplementations against androgenetic alopecia by improving peripheral blood circulation (Poller et al., 2017).

In *in vivo* trial, Song et al. (2012) reported that *L. rhamnosus* may exert a growth-promoting effect on hairs. Fermented essences prepared by using backryeoncho (*Opuntia ficus indica* var. saboten) extract and *L. rhamnosus* showed significant results in increasing hairs’ depth and number in mice without any side effect on body weight and food intake. Recently, Park et al. (2020) investigated the consequence of probiotic-enriched kimchi and cheonggukjang (a traditional Korean fermented soybean product), namely, two fermented vegetable products, on androgenetic alopecia. In this clinical study, 4 months probiotics-enriched foods interventions significantly enhanced hair counts and hair thickness, through improving blood flow without any intestinal side effect, such as diarrhea.

#### Dental Caries

*Streptococcus mutants* is the microorganism mainly contributing to dental caries. Gradual oral biofilm development is supported by different oral pathogenic bacteria, with *S. mutants* assisting to the proliferation by exopolysaccharides secretion (Lee and Kim, 2014). *Lactobacillus* and *Bifidobacterium* spp. are core probiotics promoting teeth demineralization with ultimate rise in dental plaque acidity. However, regular intake of probiotics readily reduces the *S. mutants* proliferation (Hasslöf and Stecksén-Blicks, 2020). LGG has been used for inhibiting oral colonization by cariogenic pathogens, thus reducing tooth decay incidence in children (Twetman and Keller, 2012).

According to López-López et al. (2017), oral inhabitants more actively influence oral health status, as compared to commensal gut microbiota. This is evident from *Streptococcus dentisani* activity in healthy dental plaque subjects. Such bacterium suppresses the proliferation of several oral pathogens, including *Streptococcus sobrinus* and *S. mutans*, by the production of bacteriocins. In Chile, an RCT conducted on 2- to 3-year-old children for 10 months demonstrated that a prolonged and regular intake of milk supplemented with probiotic *L. rhamnosus* SP1 significantly decreases dental caries development in preschool children (Rodríguez et al., 2016). Likewise, a 90-day-long supplementation of probiotic *Streptococcus salivarius* M18 to children provided promising outcomes against reduction in dental caries due to the strain’s ability to produce bacteriocins. Moreover, such probiotic strain synthesizes dextranase and urease enzymes that offset dental plaque and saliva acidity through colonization to oral mucosa (Di Pierro et al., 2015). Stensson et al. (2014) assessed that the regular and long-time intake of *L. reuteri* ATCC 55730 may prevent dental caries and gingivitis in children during the first year of life.

#### Osteoporosis

Osteoporosis is a complicated metabolic bone disorder in which poor bone mass, deteriorated bone tissues, and higher bone porosity lead to loss of the bone density and strength (Behera et al., 2020). Bone remodeling is a dynamic and natural process to cope with the bone damages. Osteoporosis can also occur due to bone remodeling imbalance (Collins et al., 2018). Gut microbiota play a key role in the development of osteoporosis, as the gut microbiota imbalance, particularly the increase in firmicutes, proteobacteria, and bacteroidetes populations, is one of the main causes of osteoporosis. This systematic imbalance is caused by different possible syndromes, including IBD, short bowel syndrome, systematic inflammation, hyperthyroidism, oxidative stress, abnormal somatostatin profile, non-alcoholic live fatty disease, celiac disorder, and diabetes mellitus (Nath et al., 2018). Certain drugs, including protein kinase inhibitors, heparin (anticoagulants), glucocorticoids, phenobarbital, cyclosporine, thiazolidinedione, cinacalcet, rifampicin, and doxorubicin, are also negatively associated with osteoporosis. Such drugs induce calcium, phosphorus, and vitamin D malabsorption (Aggarwal et al., 2019). Liu et al. (2019) highlighted the promising role of LGG against tenofovir-induced mandibular bone loss in *in vivo* studies. In detail, LGG inhibited the inflammation of mandible-derived mesenchymal stem cells and osteogenesis.

Nath et al. (2018) demonstrated the effective symbiotic application of lactose-based prebiotics and probiotics to retard osteoporosis by improving mineral absorption flux through the intestinal epithelium. Furthermore, conversion of insoluble inorganic salts into soluble forms, protection of intestinal mineral absorption sites, triggering of the modulation of calcium-binding proteins, and minimization of the interaction of minerals with phytic acids are the main actions reported (Nath et al., 2018). A comparative *in vivo* study conducted to evaluate the efficacy of five different probiotic strains (*L. acidophilus*, *L. reuteri*, *L. casei*, *B. longum*, and *B. coagulans*) against ovariectomized medicated bone loss indicated that probiotics efficacy is mainly strain specific, and an overall higher effectiveness was recorded for *L. acidophilus* and *L. casei* strains. Both probiotics were associated with high bone marrow concentration, bone mineral density, and bone area (global, femur, spine, and tibia) (Montazeri-Najafabady et al., 2019). Similar findings were reported by Collins et al. (2018), too: LGG and VSL3# suppressed inflammation biomarkers, i.e., TNF-α, IL-17, and receptor activator of nuclear factor kappa-B ligand (RANKL).

#### Hand, Foot, and Mouth Disease

Hand, foot, and mouth disease (HFMD) is a viral infection, common within children. Enterovirus A, i.e., Coxsackievirus-A16 (CV-A16) and enterovirus-A71 (EV-A71), are responsible for such disease. The main symptoms include oral ulcers, fever, and vesicle lesions on the hands, feet, and thigh (Xu et al., 2019). Pathologically, HFMD is considered as mild, even if it can sporadically trigger to myocarditis, meningitis, pulmonary edema, encephalitis, and lethal central nervous system problems, like acute flaccid paralysis (Zhou et al., 2016). HFMD virus is vulnerable under acidic conditions. Fu et al. (2015) identified effective HFMD antiviral activity of kombucha due to its symbiotic probiotics composition, i.e., *Saccharomyces pastorianus* and *Acetobacter xylinum*, which exhibit very high acidic activity. *In vitro* and *in vivo* intervention studies based on kombucha administration proposed its usage as potential anti-HFMD medicine. Zhu et al. (2017) evaluated the effectiveness of “Golden Bifid,” an intestinal probiotic formulation, against severe HFMD in children. Oral administration of Golden Bifid for 2 weeks to 63 severe HFMD children patients expressed impressive results. With the modulation of gut immunity, anti-inflammatory profile (IL-13, IL-4, and IL-10), and gut barrier function, intestinal probiotics can provide an efficient bio-therapy in severe HFMD.

### Immunity Disorders

#### Alleviation of Food Allergy Symptoms

In the last few decades, incidence of allergic diseases progressively increased throughout the world, with higher prevalence in western countries (Fishbein and Fuleihan, 2012). The development of oral tolerance requires association with microbes. As a matter of fact, a poor gut biodiversity during the first months of life has been associated with the development of atopic eczema. Actually, lower population levels of lactobacilli and bifidobacteria have been reported in the GIT of infants who later developed allergies (Kuitunen, 2013). Allergic diseases are disorders mainly characterized by an excessive Th2 immune response and by an unnecessary inflammatory activation of the innate immune cells. The increased incidence of allergic responses to several food products is supposed to be the result of a relative decrease in the microbial induction to the intestinal immune system during infancy and childhood (Castellazzi et al., 2013). Probiotics may improve allergies-associated symptoms by pro-T helper type 1 activation after bacterial contact. However, for optimizing results, it is necessary to define probiotic types, administration time, individual microbiota, and diet (Hajavi et al., 2019).

A study on oral immunotherapy to peanuts highlighted that participants treated with LGG were desensitized compared to the control group. In detail, the probiotics’ therapeutic effect was focused on T regulatory cells promotion (Tang et al., 2015). According to a meta-analysis encompassing 17 studies, probiotics may significantly lower the risk of infantile eczema, thus suggesting a new potential indication for probiotic use in pregnancy and infancy (Zuccotti et al., 2015). Recently, *L. rhamnosus* Lr-0601 proved to be able to modulate the balance of Th1/Th2 and Treg/Th17 in ovalbumin-sensitized mice (Song et al., 2020). In mouse model with FA induced by ovalbumin, Li N. et al. (2020) observed an abnormal gut bacterial composition, accompanied by increased immunoglobulin G, immunoglobulin E, and interleukin-4/interferon-gamma. According to the authors, *Bifidobacterium breve* M-16V may alter the gut microbiota, thus alleviating the allergy symptoms by IL-33/ST2 signaling.

#### Autoimmune Diseases

Autoimmune diseases are related to loss of the immune system’s resilience that leads to pathological immune responses. Autoimmune subjects have higher genetic biasness toward autoreactivity. Type-1 diabetes (T1D), multiple sclerosis (MS), rheumatoid arthritis (RA), and systemic lupus erythematosus (SLE) are the most common autoimmune disorders (Davidson and Diamond, 2010). Various autoimmune diseases have higher tendency toward female sex; some animal studies demonstrate the effects of the intestinal metabolism on hormonal status and of gender bias in autoimmune diseases. Gut microbiota can be a core factor to regulate immune responses and autoimmune disorders by different strategies (Yousefi et al., 2019). RA affects body joints by causing cartilages erosion, bones, and eventually joint deformities. According to a recent RCT meta-analysis on the therapeutic effect of probiotics on RA, probiotics can suppress proinflammatory cytokine IL-6—a RA biomarker—but the specific probiotic’s clinical impact is still unknown (Mohammed et al., 2017). Daily oral administration of probiotic capsules (*L. acidophilus*, *L. casei*, and *B. bifidum*) in 30 RA subjects for 8 weeks significantly reduced insulin levels, concentrations of C-reactive protein, and the disease activity score of 28 joints (Zamani et al., 2016).

Multiple sclerosis is a chronic neurological disorder that impairs the central nervous system through swelling and neural tissue damage. Probiotics may play a key role in the rehabilitation of microbial and immune balance in MS subjects (Sales-Campos et al., 2019). Clinical studies focused on MS treatment with probiotics are comparatively fewer compared to those on other autoimmune disorders. The intervention with probiotic capsules (*L. acidophilus*, *L. casei*, *B. bifidum*, and *Lactobacillus fermentum*) to 60 MS patients decreased inflammatory markers such as C-reactive protein and plasma nitric oxide metabolites. Unexpectedly, this probiotic mixture also lowered blood insulin levels, β-cell function, and total high-density lipoproteins ratio (Kouchaki et al., 2017).

T1D is related to the destruction of pancreatic b-cells, i.e., the core insulin producers. This lack of insulin production directly influences blood glucose levels (Sales-Campos et al., 2019). In one of the comprehensive cohort study entitled “The Environmental Determinants of Diabetes in the Young (TEDDY),” 7,473 Finland children from Germany, Sweden, and the United States, aged 4–10 years old and with high genetic risk of T1D, were subjected to probiotic supplementation. Results showed that the probiotic intake may decrease the occurrence of islet autoimmunity (Uusitalo et al., 2016).

The prevalence of renal and cardiovascular disease in patients with SLE is higher than in the general population. Despite strong evidence on the beneficial effects of probiotics in the development of autoimmunity in SLE, the preventive effects of probiotics in renal and cardiovascular disease in humans have not been established yet (de la Visitación et al., 2019).

### Metabolic Disorders

#### Anticancer Effects

Cancer is the second cause of death among non-communicable chronic diseases. It results in the loss of natural apoptotic cell cycle due to spontaneous accumulation of various mutagens during DNA replication process (Tomasetti and Vogelstein, 2015). Currently, cancer treatments are based on chemotherapy, radiotherapy, and immunotherapy. However, such therapeutic treatments expose cancer patients to more than one severe health side effects. Clinical and preclinical studies have been carried on in order to evaluate the efficacy of probiotics administration to manage cancer by improving the intestinal eubiosis (Vivarelli et al., 2019) (Table 3). Probiotics’ anticancer activity lies on the stabilization of intestinal microflora, strengthening of the GIT mucosal barrier, enhancing of the anti-inflammatory response, and on delaying cancer cells proliferation and metastasis development (Eslami et al., 2019). Even if probiotics are generally recognized as safe, there is still a potential risk for immune-compromised cancer patients to suffer from possible infections (Vivarelli et al., 2019).

**TABLE 3:** *In vitro* and *in vivo* effect of probiotics against cancer.

**Probiotic strain**	**Cancer type**	**Therapeutic effects**	**References**
*L. casei*	Colon carcinogenesis	Antimutagenic	Irecta-Nájera et al. (2017)
*L. fermentum*	Colorectal cancer	Anticancer	Kahouli et al. (2017)
*L. acidophilus*	Colorectal cancer	Antiproliferative	Jacouton et al. (2017)
*L. casei*, *L. johnsonii* with other strains	Colon cancer	Anticancer	Chen et al. (2017)
*L. acidophilus*, *B. bifidum*, *B. infantum*	Colorectal cancer	Antiproliferative	Kuugbee et al. (2016)
*L. rhamnosus*	Colorectal cancer	Antitumor	Gamallat et al. (2016)
*Lactococcus lactis* subsp. *lactis*	Breast cancer	Antitumor	Wu et al. (2016)
*L. crispatus, L. rhamnosus*	Cervical cancer	Antiproliferative	Motevaseli et al. (2016)
*L. crispatus, L. rhamnosus*	Breast cancer	Cytotoxic effects	Esfandiary et al. (2016)
*L. crispatus, L. rhamnosus*	Cervical and colon cancer	Cytotoxic effects	Nouri et al. (2016)
*L. acidophilus*	Lung cancer	Reduction in tumor size	Gui et al. (2015)

LGG is currently one of the most widely investigated strains for improving oncology-therapy-based side effects. LGG’s antiproliferative or antimetastatic effects have been extensively evaluated through *in vitro* models of breast, hepatic, ovary, and colorectal cancer. Besides this, LGG modulates the immune system by promoting the inhibition of tumor cells’ outgrowth (Behzadi et al., 2017; Mendes et al., 2018). Inflammation provokes cancer growth through various inflammatory-cell-based pathways in epithelial cells. The anti-inflammatory potential of probiotics can counteract cancer development under various discrete pathways, such as increased secretion of immunoglobulin A (IgA) levels, regulation of cyclooxygenase-2, and modulation of nuclear factor kappa B (NF-kB) and interferon-gamma (IFN-γ) (Shamekhi et al., 2020). Furthermore, the suppression of tumorigenesis by probiotics may be related to SCFA production, gut pathogen inhibition, intestinal enzyme activation, secondary bile acid reduction, and potential carcinogenic molecule trapping (Chong, 2014). Recently, Lagier et al. (2019) identified 11 human microbiota-based probiotic strains having the potential of triggering anticancer and anti-infectious immunity. Moreover, a specific mechanism of cecal and colonic mucus colonization and modulation of mucosal CD103+ dendritic cells seems to be involved.

#### Obesity

Obesity is considered as global epidemic resulting in abnormal or excessive fat accumulation that may be harmful for human health. Its etiology has been mainly associated with an energy imbalance along with other factors including genetic, environmental, hormonal, and infectious agents (Mirmiran et al., 2015). Gut microbiota imbalance directly influences metabolic mechanisms with ultimate results in several metabolic disorders, including obesity (León et al., 2019). *Bifidobacterium* and *Lactobacillus* spp. have been associated with improving barrier integrity. Krumbeck et al. (2018) described the symbiotic impact of galacto-oligosaccharides and *Bifidobacterium* strains on the intestinal barrier function improvement, but no synergistic effects were reported. By contrast, Ipar et al. (2015) demonstrated the symbiotic effect of *L. acidophilus*, *L. rhamnosus*, *B. bifidum*, *B. longum*, *E. faecium*, plus fructo-oligosaccharides in obese children (Table 4). After 4 weeks, probiotics-treated children experienced a significant reduction in total cholesterol, low-density lipoprotein cholesterol, and oxidative stress levels. According to Kobyliak et al. (2018), a combination of probiotics and omega-3-fatty acids proved to be effective in counteracting obesity in an *in vivo* trial. A recent investigation demonstrated that the antiobesity impact of probiotics is strain specific (Brusaferro et al., 2018). Various *in vitro* and *in vivo* studies demonstrated that probiotics, specifically *L. casei*, *L. rhamnosus*, *L. gasseri*, *L. plantarum*, and *Bifidobacterium*, i.e., *B. longum*, *B. breve*, and *B. animalis*, are widely associated with weight and fat mass index reduction (Ejtahed et al., 2019). With regard to weight control, the most promising next-generation probiotics will probably be *Akkermansia muciniphila* strains. This mucin-degrading Gram-negative bacterium, represents from 3 to 5% of the microbial community in healthy individuals and is inversely correlated with body weight in both humans and rodents (Mazloom et al., 2019).

**TABLE 4:** *In vitro* and *in vivo* effects of probiotics against metabolic disorders.

**Disease**	**Study model**	**Probiotic strain**	**Therapeutic effect**	**References**
Obesity	*In vivo humans*	*Bifidobacterium*, GOS	Symbiotic impact on the intestinal barrier function	Krumbeck et al. (2018)
	*In vivo humans*	*L. acidophilus, L. rhamnosus, B. bifidum*, *B. longum*, *E. faecium*, FOS	Reduction in total cholesterol, low-density lipoprotein cholesterol, and oxidative stress levels	Ipar et al. (2015)
	*In vivo humans*	*B. bifidum* W23, *B. lactis* W51&W52, *L. acidophilus* W37, *L. brevis* W63, *L. casei* W56, *L. salivarius* W24, *L. lactis* W19&W58	Decreased in homocysteine, TNF-α, total cholesterol, LDL, triglyceride levels	Majewska et al. (2020)
			Amelioration of total antioxidant status	
	*In vivo humans*	*B. breve* CBT BR3, *L. plantarum* CBT LP3	Reduced obesity-related markers in the probiotic group efficacy related to gut microbial enterotype	Song et al. (2020)
			Best responses in *Prevotella*-dominant enterotype	
	*In vivo humans*	*L. acidophilus*, *B. lactis*, *B. longum*, *B. bifidum*, GOS	No significant differences in body composition	Sergeev et al. (2020)
			Symbiotic increased the abundance of healthy gut bacteria	
Hypercholesterolemia	*In vivo rats*	*B. longum*, *B. lactis*, and *B. Breve, L. reuteri, L. plantarum*	Decline in total cholesterol, triacylglycerol, and in the elevation of high-density lipoprotein cholesterol decreased cholesterol-mediated proteins, including sterol regulatory element binding protein 1, fatty acid synthase, and acetyl-CoA carboxylase.	Kim et al. (2017)
	*In vivo mice*	*L. casei* pWQH01, *L. plantarum* AR113, *L. casei* LC2W	AR113 and pWQH01 induced a substantial reduction of total and LDL cholesterol, body weight, and atherogenic index	Wang et al. (2019)
	*In vivo humans*	*B. longum* BB536, red yeast rice (monacolin K, niacin, coenzyme Q10)	Improved atherogenic lipid profile	Ruscica et al. (2019)
Diabetes mellitus type-2	*In vitro*	*L. acidophilus* KLDS1.0901	Inhibition of dipeptidyl peptidase IV, retarded insulin resistance and oxidative stress	Yan et al. (2020)
	*In vitro*	*L. paracasei* 1F-20, *L. fermentum* F40-4, *B. animalis* subsp. *lactis* F1-7	Increased secretion of glucagon-like peptide 1 and peptide YY hormones, lowered TNF-α and IL-6 levels	Zhang et al. (2020)
	*In vivo*	*S. boulardii*	Controlled glycemic level, reduced inflammatory responses	Brandão et al. (2018)
	*In vivo*	*L*. *rhamnosus*, *L. acidophilus, B. bifidum*	Increased *Bacteroidetes* and declined *Firmicutes* populations	Bagarolli et al. (2017)

#### Hypercholesterolemia

Hypercholesterolemia is a metabolic syndrome mediated by abnormal serum and cellular cholesterol level (Kumar et al., 2012). Cholesterol is an important biological waxy and fat-like compound associated with lipoproteins. High serum cholesterol levels may have a direct role in the onset of many severe health disorders, i.e., coronary heart diseases, diabetes type II, hypertension, and atherosclerosis (Chien et al., 2010). Furthermore, hypercholesterolemia is considered predisposing to non-alcoholic fatty liver diseases (Wang et al., 2019). Recently, the probiotics, especially lactobacilli and bifidobacteria, is regarded as a novel therapeutic approach against hypercholesterolemia (Pavloviæ et al., 2012). Within probiotics’ cholesterol lowering mechanisms, bile salt hydrolase (BSH) enzyme is considered the most significant. In the enterohepatic circulation, BSH enzyme deconjugates the bile salts that lead to hydroxylation of conjugated glycodeoxycholic and taurodeoxycholic acids. This metabolic mechanism results in the release of glyco- and tauro-bile acids (Anandharaj et al., 2020). Besides BSH activity, 3-hydroxy 3 methylglutaryl-CoA reductase, 7-α- and 27-α-hydroxylases, and Niemann–Pick C1-like 1 protein are the proposed pathways, but a thorough understanding is still missing (Hassan et al., 2019).

Kim et al. (2017) investigated the efficacy of a probiotic mixture of *Bifidobacterium* (*B. longum*, *B. lactis*, and *B. breve*) and *Lactobacillus* (*L. reuteri* and *L. plantarum*) strains in hypercholesteremic rats. Results showed a considerable decline in total cholesterol and triacylglycerol and in the elevation of high-density lipoprotein cholesterol levels. Furthermore, in liver, probiotics decreased cholesterol-mediated proteins, including sterol regulatory element binding protein 1, fatty acid synthase, and acetyl-CoA carboxylase (Table 4). Similarly, Wang et al. (2019) compared *in vivo* the BSH activity of *L. casei* pWQH01, *L. plantarum* AR113, and *L. casei* LC2W. Strains AR113 and pWQH01 induced a substantial reduction in the total serum cholesterol, body weight, low-density lipoprotein cholesterol levels, and atherogenic index. The group treated with probiotic strain LC2W showed unsatisfactory results. According to a recent meta-analysis investigation, probiotic yogurt consumption induces a substantial reduction in total and low-density cholesterol levels among patients characterized by mild to moderate hypercholesterolemia. On the other hand, probiotic yogurt did not exert any valuable impact on high-density lipoprotein cholesterol levels (Pourrajab et al., 2020).

#### Type 2 Diabetes Mellitus

Type 2 diabetes mellitus (T2DM) is the fourth most common non-transmissible disease. Frequent weight loss, high urination, thirst, and poor foot lesions healing power are the most common symptoms, which may later lead to complications, such as coronary heart diseases, obesity, retinopathy, sexual weakness, kidney failures, and atherosclerosis (Sun J. et al., 2020). T2DM has multifactor roots, including physiological, psychological, genetic, and environmental factors (Ardeshirlarijani et al., 2019). Recent studies reported that the gut microbiota plays a crucial role in the occurrence and the management of T2DM (Vallianou et al., 2018). Probiotics can regulate insulin secretion and intestine hormonal secretion. Mainly glucagon-like peptide 1 and peptide YY can reduce fat accumulation in liver cells through L cells modulation (Will et al., 2017). Moreover, probiotics’ therapeutic potential against T2DM is based on the improvement of the gut barrier function, the increase in insulin sensitivity and critins levels, the reduction in serum lipopolysaccharides levels, as well as the oxidative and endoplasmic reticulum stress amelioration. *L. acidophilus* KLDS1.0901 is a novel promising antidiabetic probiotic strain, as demonstrated by *in vitro* dipeptidyl peptidase IV inhibitory activity along with increased blood glucose level balance and retarded insulin resistance and oxidative stress (Yan et al., 2020).

Zhang et al. (2020) screened the *in vitro* efficacy of three strains (*L. paracasei* 1F-20, *L. fermentum* F40-4, and *B. animalis* subsp. *lactis* F1-7) to understand the mechanism of insulin resistance. All probiotic strains well survived and harmonized with the gastrointestinal environment, inducing genetic modification for increasing the secretion of glucagon-like peptide 1 and peptide YY hormones, reducing the fat accumulation in liver cells, improving the glucose-uptake level, and retarding the inflammatory response through lowering the TNF-α and IL-6 levels. *In vivo* probiotic intervention study with yeast *S. boulardii* provided promising results in controlling the glycemic level and by reducing inflammatory responses and cardiovascular disorders (Brandão et al., 2018). In another *in vivo* study, probiotics *L*. *rhamnosus*, *L. acidophilus*, and *B. Bifidum* positively influenced the *Bacteroidetes* population, while *Firmicutes* population declined. Furthermore, a high-caloric diet was proven to have a negative influence on the gut microbiota by lowering the glucose tolerance and by increasing inflammation and intestinal permeability (Bagarolli et al., 2017). Kesika et al. (2019) explained that the overall efficacy of RCTs is not clear, due to some limitations, which include sample size, preparation of supplements, monitoring of intervention, sampling strategies, inconsistency in measurements, and questionnaire biasness. Authors stressed that further well-designed studies are required to understand the link between the role of the microbiome, the interaction of probiotics with the host microbiota, and the glycemic control.

### Neurodegenerative Disorders

Mental illness is referred as cognitive, emotional, and behavioral impairment. The central nervous system and brain mainly coordinate each other for normal cognitive functions. However, any abnormality in this coordination results in neurodegenerative disorders such as schizophrenia, epilepsy, dementia, autism, Parkinson’s disease (PD), and Alzheimer’s disease (AD). Inflammation and oxidation are two fundamental factors that trigger neurodegeneration through retarding normal physiological functions (Westfall et al., 2017). Currently, no direct medication is available for dementia, but medical treatments can delay this disorder (Li W. et al., 2020).

#### Parkinson’s Disease

Parkinson’s disease is the second most common complicated neurodegenerative disorder, and it occurs due to the dysfunction of the motor system that triggers impairment of dopaminergic neurons in the substantia nigra. The main symptomatic features of PD are postural variability and muscle stiffness. However, non-motor manifestations were also identified, including gastrointestinal dysbiosis, olfactory impairment, and emotional and sensorial instability (Gazerani, 2019). Levodopa is the most commonly employed drug against PD; however, this medication has certain limitations toward gastrointestinal dysbiosis and dopaminergic neuron degeneration. These side effects have promoted the interest around other potential bio-based therapies, such as gene therapy, cellular therapy, and deep brain modulation. Recently, GIT is considered as spotlight owing to its central coordination in the development of PD (Gazerani, 2019). PD patients expressed higher intestinal barrier permeability as well as accumulation of gut α-synuclein due to increasing inflammation and oxidative stress in the gut (Forsyth et al., 2011).

Preclinical and clinical intervention studies toward beneficial aspects of probiotics are yet in the narrow spectrum. Inflammation, peripheral immunomodulation, and oxidation by reactive oxygen species are vital pathological attributes of PD. Magistrelli et al. (2019) conducted an *in vitro* intervention study by using *Bifidobacterium* and *Lactobacillus* probiotics strains on isolated peripheral blood mononuclear cells obtained from PD patients. Promising and effective results were reported through reduced oxidative stress and inflammation, along with an improved gut barrier function. Moreover, a sufficient decline in the growth of gut pathogenic microorganisms, i.e., *E. coli* and *K. pneumonia* without restricting levodopa levels were observed. Most recently, Hsieh et al. (2020) *in vivo* evaluated the neuroprotective efficacy of daily and long-term consumption of probiotics on dopaminergic neurons. Results showed that regular administration of probiotics substantially decreased the motor impairments in gait pattern, balance function, and motor coordination. Probiotics can protect dopamine neurons and prevent motor dysfunction. Analogously, the novel formulation SLAB51, a combination of eight different strains of *Lactobacillus*, *Bifidobacterium*, and *Streptococcus* species, was able to counteract the detrimental effect of 6-hydroxydopamine *in vitro* and *in vivo* models of PD (Castelli et al., 2020).

#### Alzheimer’s Disease

Alzheimer’s disease is the most prevalent neurodegenerative form of dementia characterized by a gradual decline in memory, thinking, and reasoning response. The exact pathological mechanism of AD has not been confirmed yet, but there are several factors involved in the systematic pathogenesis. These include increased intestine barrier permeability, higher proinflammatory cytokines production, and impaired mitochondrial functionality, leading to excessive amounts of reactive oxygen species and oxidation (Ton et al., 2020).

Extensive scientific researches indicate that gut microbiota play a crucial role in AD neurodegenerative mechanism. Neuroactive compounds like dopamine, melatonin, serotonin, and γ-aminobutyric acid (GABA) are produced by gut microbiota (Szczechowiak et al., 2019). However, certain gut bacteria synthesize detrimental lipopolysaccharides and amyloid peptides, inducing inflammation in AD patients. Instability in gut-barrier function also contributes to neuroinflammation through contact of gut microbiota with lymphoid tissues (Jiang et al., 2017). Recently, *in vivo* studies pointed out the positive influence of oral bacterio-therapy on gut microbiota modification, which indirectly improves the neural functionality. This systematic mechanism is based on the genetic configuration enhancement that is further responsible for inflammation and neural dysfunction (Sochocka et al., 2019).

The most prevalent hallmark of AD is cognitive deficits that could be improved through the administration of lactobacilli and bifidobacteria species, as evidenced in a recent *in vivo* study (Mancuso and Santangelo, 2018). However, Sun Z. et al. (2020) carried out a comprehensive *in vivo* investigation by using probiotic *C. butyricum* to retard neuroinflammation. The probiotic intervention considerably averted cognitive impairment, neurodegeneration, microglia stimulation, Aβ accumulation, and production of tumor biomarkers (TNF-α and IL-1β). The modulations of gut microbiota and metabolic butyrate are also key phenomena observed in this study. In terms of clinical trials, Ton et al. (2020) assessed the efficacy of probiotic-based kefir intervention for 3 months in AD patients having cognitive impairments. The main biomarkers were oxidative stress levels, cytokine levels, cognitive impairment, and blood cell damage. Results showed satisfactory improvement in memory and basic cognitive functions of AD patients through the reduction in inflammation, oxidation and cell damage.

Abraham et al. (2019) proposed a combo strategy of exercise and probiotics supplementation, i.e., *B. longum*, *L. acidophilus* lysates, vitamins, and omega 3 fatty acids against AD. In such *in vivo* study, cognitive deficit reduction was related to gut microbiota regulation, improved butyrogenesis, and decreased beta-amyloid plaques level. Cerebral glucose uptake and metabolism impairment proved to be strongly associated with the pathogenesis of AD. This mechanism has been recently highlighted in mice. The oral administration of SLAB51 induced a gut microbiota prompt modification, with reference to energy metabolism and glycolysis cycle through modulation of glucose transporters and insulin growth factor-1 receptor β. Amelioration of cognitive impairment was supported by the modulation of the proteolytic cycle, reduction in β amyloid plaques, and increase in concentrations of gut microbiota neuroprotective hormones (Abraham et al., 2019).

## Conclusion

In the last two decades, extensive preclinical and clinical therapeutic studies focused on the use of probiotic strains to counteract several diseases were carried out. However, many lacking points still linger on, thus making such claims a question mark (Zucko et al., 2020). Food industry seems to be following shallow approaches instead of a holistic one regarding probiotics’ clinical efficiency. In addition, in the latest investigations, variations in results seem to be a consequence of negligence of systematic factors related to probiotics treatment protocols. Probiotics’ safety seems to be a limiting factor in most of the researches. This could be due to the widespread perception of probiotics as health-promoting agents, and this contradiction has a direct influence on probiotics’ efficacy (Zucko et al., 2020).

The probiotics’ main therapeutic potential site is believed to be the GIT, and various gastrointestinal dysbiosis are subjected to probiotics treatment. The meta-analysis of Derwa et al. (2017) mentioned uncertain probiotics’ efficacy against CD and UC, i.e., both forms of IBD. LGG and *B. animalis* subsp. *lactis* BB-12 are two of the most recognized probiotics. However, studies presently available about them are still insufficient to satisfy the confining European health claims legislation in spite of having a potential as health promoters (Flach et al., 2018). Probiotics’ interaction with host gut microbiota is another point concerning efficacy treatment. Probiotics’ beneficial aspects could be more discernable if antagonistic mechanisms against pathogenic species and growth-promoting interaction with beneficial species of gut microbiota are deeply explored (Hu et al., 2017).

## Author Contributions

MS carried on the bibliographic research. MS and NM wrote the article. MA did corrections of the manuscript. All authors thoroughly discussed and approved the final manuscript.

## Conflict of Interest

The authors declare that the research was conducted in the absence of any commercial or financial relationships that could be construed as a potential conflict of interest.
